# Fabrication and Characterization of Taro (*Colocasia esculenta*)-Mucilage-Based Nanohydrogel for Shelf-Life Extension of Fresh-Cut Apples

**DOI:** 10.3390/gels10020095

**Published:** 2024-01-25

**Authors:** Mansuri M. Tosif, Aarti Bains, Kandi Sridhar, Baskaran Stephen Inbaraj, Nemat Ali, Praveen Kumar Dikkala, Ankur Kumar, Prince Chawla, Minaxi Sharma

**Affiliations:** 1 Department of Food Technology and Nutrition, Lovely Professional University, Phagwara 144411, India; tosifmansuri444@gmail.com; 2 Department of Microbiology, Lovely Professional University, Phagwara 144411, India; 3 Department of Food Technology, Karpagam Academy of Higher Education (Deemed to be University), Coimbatore 641021, India; 4 Department of Food Science, Fu Jen Catholic University, New Taipei City 242062, Taiwan; 5 Department of Pharmacology and Toxicology, College of Pharmacy, King Saud University, P.O. Box 2457, Riyadh 11451, Saudi Arabia; nali1@ksu.edu.sa; 6 College of Food Science and Technology, Acharya NG Ranga Agricultural University, Pulivendula 516390, India; 7 Department of Basic and Applied Sciences, National Institute of Food Technology Entrepreneurship and Management, Sonipat 131028, India; 8 Department of Applied Biology, University of Science and Technology Meghalaya, Baridua 793101, India

**Keywords:** edible coating, mucilage, nanohydrogel, food preservation, cut fruits

## Abstract

Taro mucilage is a cost-effective, eco-friendly, and water-soluble edible viscous polysaccharide, which possesses diverse techno-functional properties including gelling and anti-microbial. Therefore, the objective of this study was to formulate and evaluate the efficacy of taro mucilage nanohydrogel for the shelf-life enhancement of fresh-cut apples. Taro mucilage was extracted using cold water extraction, and the yield of mucilage was found to be 2.95 ± 0.35% on a dry basis. Different concentrations of mucilage (1, 2, 3, 4, and 5%) were used to formulate the nanohydrogel. A smaller droplet size of 175.61 ± 0.92 nm was observed at 3% mucilage, with a zeta potential of −30.25 ± 0.94 mV. Moreover, FTIR data of nanohydrogel revealed the functional groups of various sugars, uronic acids, and proteins. Thermal analysis of nanohydrogel exhibited weight loss in three phases, and maximum weight loss occurred from 110.25 °C to 324.27 °C (65.16%). Nanohydrogel showed shear-thinning fluid or pseudo-plastic behavior. Coating treatment of nanohydrogel significantly reduced the weight loss of fresh-cut apples (8.72 ± 0.46%) as compared to the control sample (12.25 ± 0.78%) on the 10th day. In addition, minor changes were observed in the pH for both samples during the 10 days of storage. Titrable acidity of control fresh-cut apples measured 0.22 ± 0.05% on day 0, rising to 0.42 ± 0.03% on the 10th day, and for coated fresh-cut apples, it was observed to be 0.24 ± 0.07% on the 0th day and 0.36 ± 0.06% on 10th day, respectively. Furthermore, the total soluble solids (TSS) content of both control and coated fresh-cut apples measured on the 0th day was 11.85 ± 0.65% and 12.33 ± 0.92%, respectively. On the 10th day, these values were significantly increased (*p* < 0.05) to 16.38 ± 0.42% for the control and 14.26 ± 0.39% for the coated sliced apples, respectively. Nanohydrogel-coated fresh-cut apples retained antioxidant activity and vitamin C content as compared to the control sample. Taro mucilage nanohydrogel-based edible coating showed distinct anti-microbial activity against psychrotrophic, aerobic, and yeast molds. In summary, taro mucilage nanohydrogel can be used as a cost-effective natural coating material for the shelf-life enhancement or freshness maintenance of fresh-cut apples.

## 1. Introduction

Fresh-cut fruits and vegetables are categorized as perishable food and are susceptible to fast deterioration or spoilage due to various environmental factors including temperature, gases, humidity, and microorganisms [1]. Infected foods with microbial contamination increase the risk of illnesses that can range from acute neurological disorders, cancer, and diarrheal diseases to eventual death [2]. Therefore, researchers and industrial innovators are focusing on food waste, as food waste emerges as the primary threat to food security. Globally, one-third of processed food products are wasted, adding a staggering 1.30 billion tons each year [3]. The challenges faced in the food supply system are further intensified by the rising demand for food products. Moreover, the threat of contamination by pathogenic microbes remains extensive. The harmful consequences of microbial growth in food are apparent in unfavorable alterations to flavor, color, appearance, and texture. Over the past few years, several efforts or techniques have been employed toward developing novel food preservatives sourced from natural origin without side effects on human health and the environment [4,5]. Furthermore, plant-derived biopolymers offer several advantages over synthetic materials, including low cost, biodegradability, biocompatibility, and adaptability to biochemical and chemical processes. The combination of food-grade edible coating materials with specific additives is known to enhance the shelf-life and extend the ease of food consumption [6]. On the other hand, nanotechnology plays a significant role in the development of carriers to transport specific compounds such as plant extracts, vitamins, essential oils, and drug delivery. They present a promising material due to the smaller size of particles which enhances the mechanical resistance and the controlled release of the natural product [7]. Consequently, nanotechnology encompasses systems of submicron size (<1000 nm), particularly those ranging from 100 to 500 nm. The increased surface area per mass, in comparison to larger particles of the same chemical composition, imparts greater stability and biological activity to nanosystems. This allows for the incorporation of hydrophobic and/or active substances without significantly altering appearance or transparency, thereby preserving the visual characteristics and extending the shelf life of foods. Nanocarriers including nanoemulsions and nanohydrogel are potentially used as a cost-effective and eco-friendly edible coating material for the shelf-life enhancement of food products [8]. 

Gums and mucilage are water-soluble hydrocolloids that can be extracted from various natural sources. Mucilage has excellent gelling and water-holding properties in the presence of water. Comprising primarily of monosaccharides, these compounds are often bound with organic acids [9]. In addition, they have remarkable techno-functional properties and distinct health benefits. Therefore, mucilage is an excellent addition to food formulations and is used for food preservation due to its numerous advantageous properties. They contribute emulsifying, thickening, and rheology-modifying characteristics, enhancing the overall texture and consistency of foods and possessing naturally anti-microbial efficacy [10]. These diverse features of the mucilage make them potential sources of edible coating material for the shelf-life enhancement of food products [11]. 

Taro (*Colocasia esculenta*) is a tropical root crop and is famous due to its abundant amount of carbohydrates, especially starch and mucilage. The chemical composition of taro mucilage comprises carbohydrates (arabinose, xylose, mannose, glucose, and galactose) and proteins (major amino acids including lysine, tryptophan, cysteine, isoleucine, and leucine) [12,13]. This broad-spectrum nutritional composition increases the demand for taro mucilage. However, currently, very limited applications are employed for the utilization of taro mucilage as a cost-effective material. Therefore, the present approach emphasized the fabrication of taro-mucilage-based nanohydrogel. Also, the efficacy of the prepared nanohydrogel was evaluated for the shelf-life enhancement of the fresh-cut apples. 

## 2. Results and Discussion

### 2.1. Taro Mucilage Yield

Taro mucilage extracted using cold water extraction was a cost-effective and sustainable approach to extract the mucilage. The yield of taro mucilage was calculated to be 2.95 ± 0.35%. The yield of mucilage can be varied based on the maturity of the taro rhizomes and geographical conditions. Herein, the existence of starch in taro mucilage can be regarded as a significant impurity that adversely impacts its quality and does not contribute to effective emulsification properties [14]. Therefore, the cold-water extraction method was employed for the extraction of starch-free mucilage. In addition to the extraction methods, several factors, including the degree of branching, molecular size, distribution, structural linkages, maturity stage, the presence of hydrophobic components, and monomeric compositions, play a crucial role in influencing the functional properties of taro mucilage. A similar result was observed by Andrade et al. [14]. They extracted taro mucilage using five different methods and conditions including cold water extraction. In their study, the yield of mucilage was found to be 1.33% which was similar to the present study. However, starch existed in the other four methods. These results were directly influenced by the functional properties of the mucilage. 

### 2.2. Characterization of Nanohydrogel

#### 2.2.1. Functional Group Determination

**Figure 1:**
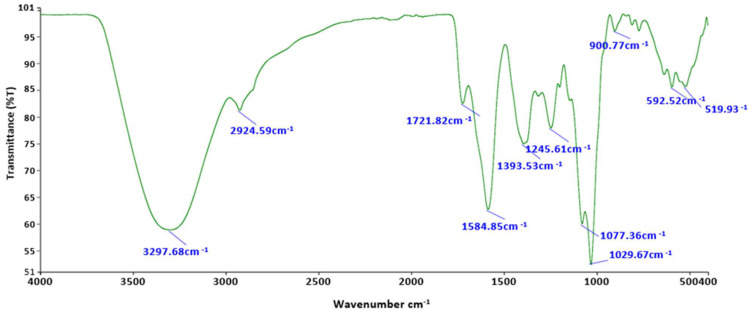
Functional group evaluation of taro mucilage nanohydrogel.

The presence of various functional groups in the taro mucilage nanohydrogel was determined through FTIR analysis, with the corresponding data represented in Figure 1. Herein, various sugars, proteins, and uronic acid groups were found. At the wavenumber of 3297.68 cm^−1^ (-OH), stretching vibration was confirmed due to the presence of the hydroxyllic (-OH) group of polysaccharides. It has been believed that taro mucilage has excellent water-holding and oil-binding capacity due to the presence of hydrocyllic groups of various sugars [15]. When taro mucilage nanohydrogel comes into contact with water (H_2_O), it creates strong hydrogen bonding between sugar molecules and hydrogen atoms. Additionally, -CH stretching vibration was observed at the wavenumber of 2924.59 cm^−1^. Taro mucilage was found to contain a certain amount of proteins, as evidenced by FTIR analysis at a wavenumber of 1584.85 cm^−1^ (associated with N-H bending). The emulsifying capability of the taro mucilage was attributed to the presence of different sugars and protein fragments. In the FTIR spectra, the wavenumber between 800 and 1300 cm^−1^ is referred to as the fingerprinting area of the carbohydrate. In this context, a band was observed at 1029.67 cm^−1^, corresponding to the C-O stretching vibration of glucose. The absence of starch was confirmed in the nanohydrogel sample because in the FTIR spectra, a mostly starchy compound band can be found below the 925 cm^−1^ wavenumber that is an indicator of the starchy compounds, and it mainly contains -CH and -NH stretching as well as linkage vibrational modes of C-C and C-O [16]. Similar results were proved in a study conducted by Andrade et al. [14], where taro mucilage was extracted using five different conditions. However, starch-free taro mucilage exhibited by employing cold water extraction and FTIR data proved similar functional groups in mucilage structure. In the present study, FTIR data confirmed the presence of protein molecules in the starchless taro mucilage nanohydrogel, directly influencing the techno-functional characteristics of the mucilage. The primary sugar in the taro mucilage nanohydrogel was identified as galactose, confirmed at a wavenumber of 1029.67 cm^−1^, which corresponds to C-O vibration. Each peak in the FTIR spectrum holds significance for the functional and structural properties of the mucilage.

#### 2.2.2. Thermal Analysis

**Figure 2:**
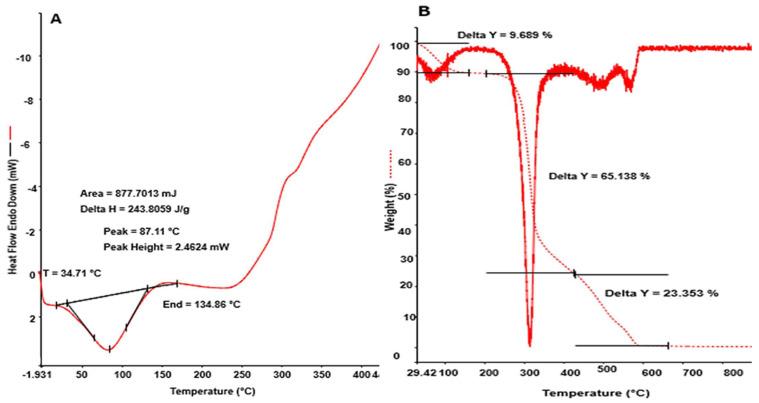
Thermal stability (**A**) and thermogravimetric analyses (**B**) of the taro mucilage nanohydrogel.

The thermal stability of taro mucilage nanohydrogel was evaluated using differential scanning calorimetry (DSC) and thermogravimetric analysis (TGA). The occurrence of physical and chemical changes during the heating of nanohydrogel is presented in Figure 2A. Two endothermic peaks were observed during the thermal analysis. The first peak was observed at 34.71 °C which was attributed to the water loss and breakdown of O-H bonds in the mucilage backbone [17]. Meanwhile, the second peak was observed at 87.11 °C with a heating enthalpy of ∆243.80 J/g. Herein, nanohydrogel absorbs the heat, and due to that, the breakdown of the chemical bonds (C-O and -COO) of mucilage occurs [18]. Furthermore, TGA deals with the gradual change in the mass of nanohydrogel with increasing temperature. The weight of the nanohydrogel was evaluated at the high temperature of 30 °C to 950 °C. Weight loss occurred in three phases. Minor weight loss (∆Y = 9.689%) occurred at 98.65 °C which was due to the phase transition of the nanohydrogel and attributed to the loss of moisture content. In the second phase, maximum weight loss occurred (∆Y = 65.168 %) at 246.79 °C to 408.19 °C, as shown in Figure 2B. The chemical reaction also takes place between this temperature due to the breakdown of monomeric units of mucilage-based nanohydrogel. In the third phase, the remaining residues including ash and other organic compounds were 23.35%. Consequently, the prepared nanohydrogel has higher heat stability than the chitosan-based nanohydrogel. Shah et al. [19] prepared chitosan and carboxymethyl cellulose-based multi-functional bioactive nanohydrogel. TGA data revealed the maximum weight loss of hydrogel at 300–315 °C (∆Y = 95%) attributed to the degradation of the polysaccharide. 

#### 2.2.3. Rheological Behavior

**Figure 3:**
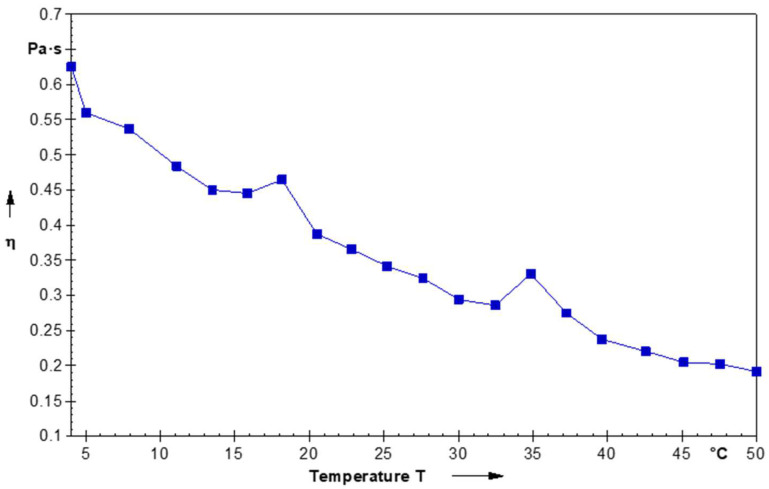
Rheological behavior of taro mucilage nanohydrogel.

Temperature affects the swelling capacity of taro mucilage gel and also forms a three-dimensional gel-like network. Taro mucilage nanohydrogel showed the shear-thinning behavior as the decrease in the viscosity with increasing the temperature from 0 to 50 °C. During the heating condition, the phase transition of material converts from a liquid to a gel state with the effect of mass transfer. A major aspect of the phase transition changes the viscoelastic property and develops a solid-like structure. As the shear rate increased, the taro mucilage coating solution demonstrated pseudo-plastic behavior, also known as shear-thinning fluid behavior, as shown by a drop in viscosity (Figure 3). The viscosity of the nanohydrogel was decreased due to the deformation and alignment of the nanohydrogel droplets when exposed to the temperature. At lower temperatures (19 °C) and (35 °C), molecules of the mucilage exhibit a more random orientation, leading to stronger interparticle interactions and increased flow resistance, consequently increasing the apparent viscosity of the nanohydrogel [20]. The shear-thinning behavior of nanohydrogel states the increasing viscosity with the shearing applied temperature surpasses the rate of entanglement formation. This behavior in the disentanglement of mucilage molecules depletes the network and leads to a decrease in viscosity. With an elevation in shear rate, one anticipates the polymer molecules to disentangle and orient themselves in the direction of flow, thereby minimizing resistance and causing a decrease in viscosity [17]. In addition, the rheological (viscoelastic) characteristics of the hydrogel are usually evaluated to check the techno-industrial importance in a specific application. Furthermore, the rheological property of mucilage can be affected by the interaction between different polymers [18]. A similar observation was found in the changes in the viscosity of guar gum. Guar gum gel exhibited non-newtonian, shear-thinning behavior, and the apparent viscosity was decreased with the increasing shear rate. The viscosity of polysaccharide-based hydrogel depends upon the molar mass while synergistic interactions are determined by the different ratios of existing sugars and their structural evidence [21]. 

#### 2.2.4. Droplet Size and Zeta Potential

The droplet size of the nanohydrogel formulated using different taro mucilage concentrations (1, 2, 3, 4, and 5%) and their zeta potential are shown in Table 1. The lower droplet size (175.61 ± 0.92 nm) was found in the 3% taro mucilage concentration, and a higher droplet size (245.35 ± 0.58 nm) was in the 1% mucilage, respectively. Taro mucilage is a highly viscous substance that contains protein and sugar compounds, and it increases the swelling capacity of the hydrogel when in contact with water. This swelling is attributed to the presence of hydroxyl groups within the mucilage, which interact with the hydrogen atoms of water (H_2_O), leading to the formation of strong hydrogen bonds within the structure. As a result, mucilage effectively binds or entraps water within its structure. Meanwhile, 211.48 ± 1.24 nm, 199.76 ± 0.75 nm, and 186.19 ± 0.37 nm droplet sizes were observed with the −15.79 ± 0.43 mV, −12.81 ± 0.71 mV, and −19.46 ± 0.37 mV zeta potentials at the 2, 4, and 5% mucilage concentrations, respectively. Mucilage is a long-chain polymeric polysaccharide that can become entangled with each other in the presence of water or any suitable solvent. At lower concentrations, these chains are more dispersed and have the ability to form longer particles and aggregation due to the weaker interactions within the particles. Meanwhile, as the concentration increases, the chances of entanglement also increase, resulting in a smaller and more compact structure. However, mucilage exhibited excellent water-holding capacity and gel forming due to the interaction between the different hydrophilic and hydrophobic compounds. Therefore, after a certain limit, the gel-forming capacity of mucilage nanohydrogel is reduced due to the changes in the concentration of mucilage. Likewise, the electric charge (zeta potential) of nanohydrogel is highly dependent on the concentration of the polysaccharide and the type of polysaccharide [22].

### 2.3. Physicochemical Properties of Fresh-Cut Apples

**Figure 4:**
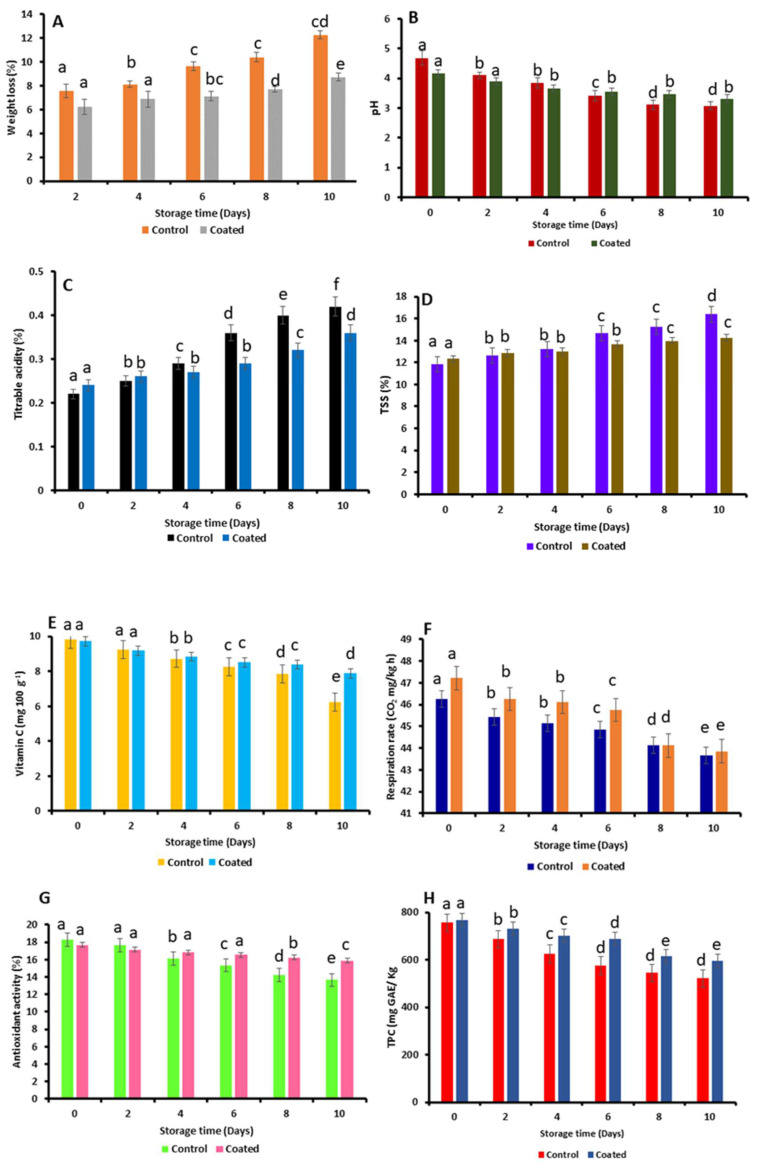
Physicochemical properties of coated and uncoated apples. (**A**) Weight loss, (**B**) pH, (**C**) titrable acidity, (**D**) TSS, (**E**) vitamin C, (**F**) respiration rate, (**G**) antioxidant activity, and (**H**) TPC. The error bars represent the means with standard error of the three replicate samples, and various lowercase letters above the error bar show the significant difference (*p* ≤ 0.05) between coating treatments.

The effects of taro mucilage nanohydrogel edible coating on the pH and weight loss of fresh-cut apples are shown in Figure 4A,B, respectively. Nanohydrogel effectively reduced the weight loss for coated fresh-cut apples as compared to the control sample. In fruits and vegetables, the main elements that directly affect their physicochemical properties are water activity, moisture content, and respiration rate. The coated apples lost significantly less weight (8.72 ± 0.46%) than the control apples, which lost the most weight (12.25 ± 0.78%) on the tenth day. These results suggest that the taro mucilage nanohydrogel-based edible coating plays a significant role in the prevention of weight loss in fresh-cut apples. Coated nanohydrogel acts as a protective layer that creates a barrier and helps to reduce the weight (moisture) loss from the cut-fruit surface. Also, it decreases the water vapor transmission rate by forming a thin film layer on the surface which can significantly minimize the weight loss of the fruits [23]. Similarly, an edible coating material prepared from a combination of aloe vera and carboxymethyl cellulose decreased weight loss in fresh-cut apples and increased the food product’s shelf life by protecting it from extrinsic and intrinsic influences. Tosif et al. [19] reported a similar result in a prior investigation. The pH of the control apples decreased significantly during the duration of the experiment, falling from 4.68 ± 0.37 on the 0th day to 3.08 ± 0.46 on the 10th day. On the other hand, the pH of the taro-mucilage-coated apples decreased slightly during the study, rising from 4.16 ± 0.55 on day 0 to 3.32 ± 0.29 on day 10. 

In addition, titrable acidity and total soluble solids (TSS) are important factors to consider when evaluating the overall quality of food items. Figure 4C,D show the titrable acidity and TSS of both coated and uncoated fresh-cut apples. This suggests that constant weight loss leading to decreased dehydration is responsible for the slight rise in TSS during storage in edible-coated apples. The increasing trend in the TSS was observed due to the breakdown of complex carbohydrates into simple sugars of apples. For control fresh-cut apples, the titrable acidity was 0.22 ± 0.05% on the 0th day and increased to 0.42 ± 0.03% on the 10th day. For coated fresh-cut apples, the titrable acidity was 0.24 ± 0.07% on day 0 and 0.36 ± 0.06% on day 10, respectively. The physicochemical parameters of fresh-cut apples were significantly affected by the application of taro mucilage nanohydrogel coating. An edible coating of taro mucilage nanohydrogel can act as a surface barrier of apples that potentially helps retain the natural acidity present in the cut apples. This can lead to minor changes in the acidity as compared to the control sample. Furthermore, the coating layer of nanohydrogel reduces the degradation of acids during the 10-day storage period. This can contribute to maintaining the acidity level of the cut apples. Notably, fresh-cut apples coated with taro mucilage nanohydrogel demonstrated a remarkable impact on the overall physicochemical properties. The gel itself forms a protective barrier on the food surface, guarding against various environmental factors. Furthermore, on the 0th day, the TSS of the coated fresh-cut apples and the control apples was 12.33 ± 0.92% and 11.85 ± 0.65%, respectively. On the 10th day, these values were increased up to 16.38 ± 0.42% for the control and 14.26 ± 0.39% for the coated apples. The TSS level and overall sensory attributes of the apples depend on the maturity stage and the variety type of fruits [24]. The increase in the TSS value was attributed to the breakdown of the complex sugars into the simpler sugar structure. Another reason is that taro mucilage is composed of several natural sugars; therefore, sugar-based coating material might maintain the sugar level of the cut apples. 

**Figure 5:**
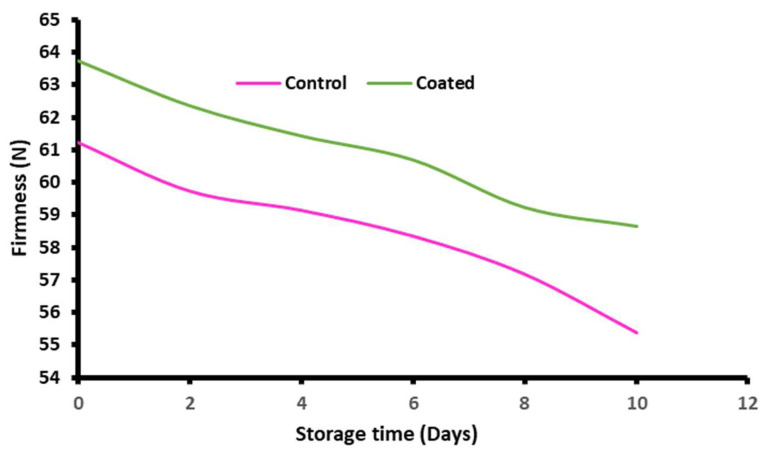
Firmness of control and fresh-cut apples coated with taro mucilage nanohydrogel.

Ascorbic acid and the antioxidant activity of control and coated fresh-cut apples were evaluated, and their results are shown in Figure 4E,G, respectively. Herein, it can be observed that taro-mucilage-based nanohydrogel has a great ability to protect the bioactive compounds and vitamins. The ascorbic acid content of the control apples was found to be 9.83 ± 0.88 mg/100 g^−1^ on the 0th day and decreased to 6.25 ± 0.61 mg/100 g^−1^, and for coated apples, it was found to be 9.72 ± 0.48 mg/100 g^−1^ on the 0th day and 7.88 ± 0.28 mg/100 g^−1^ on the 10th day. Taro mucilage nanohydrogel edible coatings can act as a protective barrier, helping to retain the ascorbic acid content in fresh-cut apples. Coated nanohydrogel on cut apples prevented the degradation of ascorbic acid by reducing the exposure to light and oxygen. Ascorbic acid is highly affected by various environmental factors when fruits are exposed to air, and it is sensitive to oxidation. Thereby, taro mucilage has natural antioxidant properties, which help to minimize the oxidation of ascorbic acid [25]. Antioxidants and ascorbic acids (vitamin C) are the health-promoting components that can help prevent free radical damage to cells. It is believed that foods containing a higher ascorbic content can help to maintain cartilage, bones, blood vessels, and healthy skin. Moreover, tannins, phenols, lignans, and flavonoids are naturally occurring antioxidants originating from plant sources [26]. Fresh-cut apples coated with taro mucilage nanohydrogel effectively retained the total phenolic content (TPC) of the apples, as shown in Figure 4H. For the control sample, total TPC was observed at 756.22 mg (gallic acid equivalents) GAE/kg on the 0th day which was significantly decreased to 521.08 mg GAE/kg on the 10th day. In addition, for nanohydrogel-coated fresh-cut apples, it was 768.18 mg GAE/kg on the 0th day and 594.86 mg GAE/kg on the 10th day. Taro mucilage nanohydrogel helps to retain the total phenolic content in fruits. Phenolic compounds are often susceptible to degradation due to factors such as enzymatic and oxidation activities, and coatings may slow down these processes [27]. Furthermore, the respiration rate of the control and coated fresh-cut apples was found to be 46.25 mg/kg h and 47.22 mg/kg h on the 0th day, and it decreased to 43.65 mg/kg h and 43.85 mg/kg h on the 10th day, as shown in Figure 4F. Coated nanohydrogel offers protection against external factors including contaminants and physical damage. This protection may indirectly influence the respiration rate by preventing stress responses that could accelerate respiration [28]. Meanwhile, the antioxidant activity of the control and coated cut apples was observed at 18.25 ± 0.36% and 17.68 ± 0.58% on the 0th day which was significantly decreased to 13.64 ± 0.75% and 15.85 ± 0.18% on the 10th day, respectively. Moreover, the prepared nanohydrogel coating has inherent antioxidant properties, contributing to the preservation of phenolic compounds by reducing oxidative stress, and can help maintain the overall antioxidant activity of the cut fruits [29]. Figure 5 displays the firmness value of the control and coated fresh-cut apples. Firmness is a textural characteristic to assess fruit quality because fresh, undamaged fruits and vegetables have a higher firmness than stored, residual, and damaged ones. The results of the firmness showed an effective impact on the coated apples as compared to the control apples. The firmness value of the control sample was found to be 55.39 N on the 0th day which was decreased to 55.08 N on the 10th day, and for coated apples, it was found to be 63.73 N on the 0th day and 58.64 N on the 10th day, respectively. Mucilage-based nanohydrogel coating protects the cut apples against physical damage and mechanical and bruising injuries. Overall, nanohydrogel coating showed comparable firmness values to those of the control sample by minimizing the damage. Also, it helps to preserve the structural integrity of the cut apples [30].

### 2.4. Microbial Analysis 

**Figure 6:**
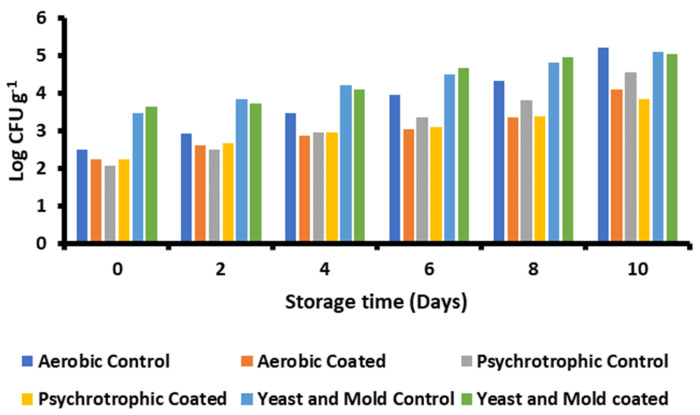
Antimicrobial efficacy of fresh-cut apples against bacteria and fungus.

Taro-mucilage-based nanohydrogel significantly enhanced the shelf-life of fresh-cut apples by inhibiting microbial growth during the 10-day storage period. It was observed that the microbial growth of the control apples was significantly higher than coated apples, as shown in Figure 6. Furthermore, the microbial growth of psychrotrophic and aerobic bacterial counts for edible-coated fresh-cut apples was significantly lower on the 0th day, 2.25 log CFU g^−1^ and 2.24 log CFU g^−1^, respectively, which was increased to 3.85 log CFU g^−1^ and 4.09 log CFU g^−1^ on 10th day. 

**Figure 7:**
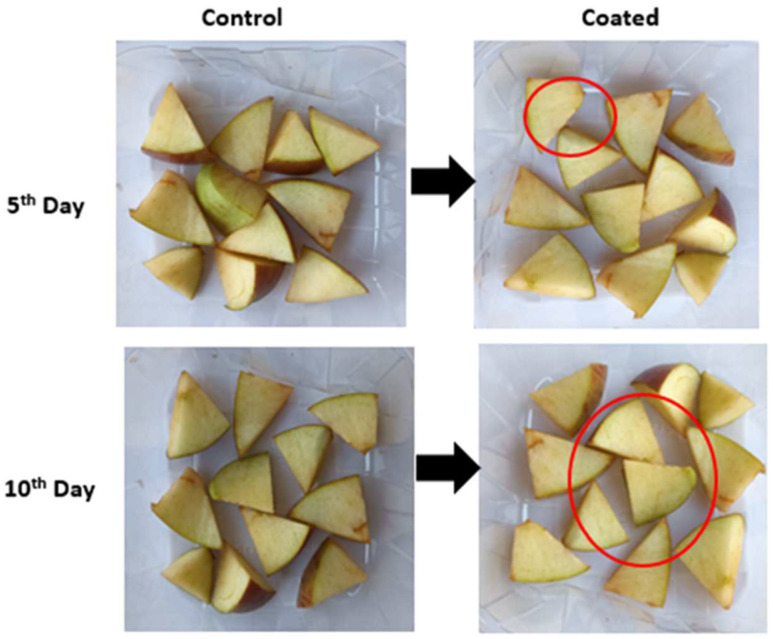
The visual appearance of TMCG-coated and uncoated cut apples.

The psychrotrophic and aerobic bacterial counts for the control apples were found to be 2.08 log CFU g^−1^ and 2.50 log CFU g^−1^ on the 0th day, respectively. By day 10, these values were increased to 4.55 log CFU g^−1^ and 5.22 log CFU g^−1^, respectively. Because uronic acids are present in mucilage, it demonstrated excellent anti-microbial activity in the present study. Taro mucilage nanohydrogel has the capacity to extend the shelf life of fresh-cut apples by protecting them from physical, chemical, and biological hazards, which are major sources of microbial spoilage (Figure 7) [31]. However, the counts of mold and yeast for the edible-coated apples were 3.65 log CFU g^−1^ on day 0 and 5.05 log CFU g^−1^ on day 10, respectively. The counts for the control group were 3.46 log CFU g^−1^ on day 0 and increased to 5.09 log CFU g^−1^ on day 10. Bacteria’s ability to adhere to host tissues and cells can be hindered by taro mucilage nanohydrogel, which in effect prevents the bacteria from binding to apple surfaces [32]. The early phases of bacterial establishment and infection are disrupted by this intervention. Furthermore, by adhering to lipopolysaccharides or peptidoglycans, nanohydrogel can disrupt bacterial cell membranes, exposing their integrity. This interference results in the release of cellular contents and eventual cell lysis [33]. 

## 3. Conclusions

The present findings proved that taro mucilage nanohydrogel has an excellent synergistic effect on the edible-coated fresh-cut apples during the 10 days of storage. Nanohydrogel coating material retained the bioactive compounds including antioxidants, phenols, and ascorbic acid (vitamin C) and overall maintained the tissue softening of the apples. Also, nanohydrogel coating material reduced the microbial growth which led to the enhancement of the shelf life of fresh-cut apples. Therefore, these findings may be a useful way to maintain the freshness of processed fruits and vegetables as a cost-effective natural alternative ingredient. Moreover, taro is a novel mucilage source of under-utilized crops that can be a great alternative to synthetic material due to its low cost, eco-friendly properties, and remarkable anti-microbial efficacy. However, there is a need to highlight such sources of mucilage, and more research is needed for the utilization of taro mucilage in different food and non-food applications. The formulation of a nanohydrogel-based coating solution showed excellent commercial features like preventing environmental factors and improving the overall quality of the cut apples for approximately up to 10 days. 

## 4. Materials and Methods 

### 4.1. Materials

Fuji cultivator of apples was purchased from the local fruit market of Shimla, Himachal Pradesh, India. Taro rhizomes were obtained from the local vegetable market of Jalandhar Punjab, India. Normal-sized apples were selected to conduct all experiments. For physicochemical analysis of fresh-cut apples, various chemicals and reagents such as Folin–Ciocalteu reagent, 2,2-diphenyl-1-picryl-hidrazil (DPPH), and 2,6-dichlorophenolindophenol were obtained from ACS chemicals, Ahmedabad, Gujarat, India. For microbial analysis, nutrient agar and potato dextrose agar were procured from *Chaitanya Agro Biotech Pvt. Ltd.,* Mumbai, Maharashtra, India.

### 4.2. Sample Preparation

Fresh fuji apples were washed with running tap water and stored at ambient temperature (27 °C) for further analysis and application. Before the coating treatment, the apples were cut into a uniform size (4 × 6 cm) and shape. Medium-sized taro rhizomes (30 cm length and 15 cm diameter) were rinsed with deionized water, followed by peeling, and were then cut into uniform pieces (round shape). To prevent contact with the environmental factors including oxygen and temperature, eight apples were selected and processed for further analysis. The average weight of the apple was found to be 254.84 ± 2.21 g. 

### 4.3. Methods

#### 4.3.1. Extraction of Taro Mucilage

The cold-water extraction method recommended by Andrande et al. [14] was used to extract the taro mucilage with some modifications. Briefly, 100 g of taro rhizomes was sliced into uniform pieces and added to 250 mL of cold distilled water. The material was homogenized for 5 min at 6000 rpm to prepare the slurry. After filtering the slurry through muslin fabric, centrifugation was performed for 15 min at 4 °C. The supernatant was collected, and after 24 h of drying at 50 °C in a hot-air oven, a homogeneous powder was obtained. For future use, the powdered mucilage was kept in an airtight container. Equation (1) was used to calculate the mucilage yield.

(1) $$Mucilage\;yield\left(\%\right)=\left[\left(\frac{Total\;weight\;of\;dry\;mucilage\;(g)}{Total\;amount\;of\;sample\;(g)}\times 100\right)\right]$$

#### 4.3.2. Development of Nanohydrogel

Different concentrations of taro mucilage (1, 2, 3, 4, and 5%) were used to prepare the nanohydrogel according to the procedure outlined by Thakur et al. [33]. Herein, the low-energy method was employed for the fabrication of nanohydrogel. Canola oil was used as the oil phase, and tween 80 was used as the ionic surfactant. Briefly, above-mentioned concentrations of mucilage were dissolved in 100 mL of double-distilled water with continuous stirring using a magnetic stirrer at 1500 rpm. Subsequently, 2 mL of canola oil (Jivo, Mumbai, Maharashtra, India) and 800 μL of tween 80 were added to the aqueous phase and thoroughly mixed at 1200 rpm for 120 min on a magnetic stirrer (Remi, Delhi, India). The formation of a milky white solution indicated successful emulsion formation. In the second step, an emulsion-based taro mucilage nanohydrogel was prepared through covalent bonding with pectin serving as the cross-linker. Different concentrations (1, 2, 3, 4, and 5% of mucilage) and 2 mL of glycerol were used as a binding agent. These materials were introduced to the emulsion, and polymerization was initiated using continuous stirring at 1200 rpm for 2 h on a magnetic stirrer (Remi, Delhi, India). The emergence of a 3D gel-type structure confirmed the successful formulation of the nanohydrogel. Finally, the samples were preserved in glass vials at a refrigerated temperature for subsequent analysis. The selection of nanohydrogel was carried out based on the droplet size of nanohydrogel. For coating treatment, fresh-cut apples (50 g) were dipped into 200 mL of 3% nanohydrogel coating solution for 3 min and stored in the plastic tray in refrigeration condition (4–7 °C). Meanwhile, 50 g of fresh-cut apples was kept as a control sample. Furthermore, the storage study of the coated and control cut apples was performed at different time intervals for 10 days.

#### 4.3.3. Characterization of Nanohydrogel

##### Fourier Transform Infrared Spectroscopy (FTIR) Analysis

The existence of functional groups was assessed in the taro mucilage nanohydrogel using FTIR, and the spectra were recorded using Attenuated total reflectance (A.J Instruments Private Limited, Delhi, India). Taro mucilage nanohydrogel (10 µL) was subjected to the ATR diamond plate surface, and runs were performed in triplicates in the wavenumber range of 4000 to 400 cm^−1^. The transmittance was collected using the built-in Spectrum 10 software.

##### Thermal Analysis

The thermal analysis of the taro mucilage nanohydrogel was performed to study the physical and chemical reactions occurring within the sample using differential scanning calorimetry (Perkin Elmer 6000, Tokyo, Japan). The temperature was increased from 0.01 °C/min to 100 °C/min, and the experiment was conducted in a controlled atmosphere of 99.999% nitrogen. Taro mucilage nanohydrogel (10 mL) was injected into aluminum DSC pans (Al crimp Pan C.201–52943). Under a nitrogen atmosphere, the measurements were conducted throughout a temperature range of 25 °C to 400 °C using a heating rate of 10 °C per minute.

Thermogravimetric analyses (TGAs) were performed to measure the weight loss of nanohydrogel during the heating process using a Shimadzu TGA-50 (Shimadzu Corporation, Kyoto, Japan). Samples were placed in the balancing system and heated in a nitrogen environment between 25 and 600 °C at a heating rate of 10 °C per minute. At least two repetitions of each kind were made for every measurement.

##### Rheological Behavior

The rheological behavior of taro mucilage nanohydrogel was assessed in accordance with Nesrinne et al.’s [34] method using a digital rheometer (MARS III, ThermoFisher Scientific, Newington, NH, USA) fitted with a parallel plate configuration (35 mm radius). Prior to performing rheological testing, the nanohydrogel was thoroughly mixed and centrifuged for 10 min at 4000 rpm in order to remove residual bubbles. The nanohydrogel was then placed on the sample plate and exposed to stress-sweep studies, which were conducted at a temperature of 5–50 °C and covered a stress range of 0.1 to 1000 Pa at a frequency of 1 Hz. Using a gap size of 1.0 mm, the shear viscosity was measured in rotational ramp mode throughout a range of shear rates.

##### Droplet Size and Zeta Potential

The analysis of droplet size and size distribution for the taro mucilage nanohydrogel was carried out using a Zetasizer Nano ZS90 instrument from Malvern Instruments Ltd. (Malvern, UK). For the sample preparation, taro mucilage nanohydrogel (1% *v*/*v*) was prepared by suspending it in 20 mL double-distilled water, and the sample was then subjected to ultrasonication using the probe ultra sonicator to avoid the extra bubbles (Samarath Electronics, Thane, India) for 10 min. The analysis was taken in triplicates using fresh cuvettes for each trial. 

### 4.4. Physicochemical Properties of Fresh-Cut Apples

#### 4.4.1. Weight Loss and pH

The weight loss of fresh-cut apples with and without nanohydrogel coating was measured according to the method followed by Nandane et al. [35]. In brief, samples were weighed (5 g each coated and control) and placed in glass Petri plates in the refrigeration condition (4–7 °C). Throughout a 10-day storage period, the weight loss was measured at different time intervals (on the 2nd, 4th, 6th, 8th, and 10th day). Moreover, the pH of fresh-cut apples was measured according to the method followed by Khorram et al. [27]. A digital pH meter (Forbes Marshall, Pune, India) was used to measure the pH of coated and uncoated apples. Samples (20 g each) were homogenized in 50 mL of deionized water using a high-speed homogenizer, and slurry was further used for the pH determination.

#### 4.4.2. TSS and Acidity

The total soluble solids (TSS) and acidity of fresh-cut apples, both coated and uncoated, were measured by following the method proposed by Tokatlı et al. [36]. Fresh-cut apples (50 g each) were added to the 100 mL of distilled water, and a laboratory hand blender was used for the preparation of slurry. This slurry was then employed to measure TSS and titratable acidity. Whatman filter paper 1 was used to filter the resultant slurry in order to assess TSS. The filtrate was then further purified by centrifugation at 7000 rpm for 15 min. The TSS of apples was determined using a digital reflectometer (Mettler-Toledo, Mumbai, India). Titrable acidity of cut apples was evaluated by the titration of the prepared slurry using 0.1 M NaOH solution and titration to an end point of pH 8.2 as indicated by a pH meter (Myron, Carlsbad, CA, USA). Phenolphthalein was used as an indicator to study the endpoint of the titration process [37].

#### 4.4.3. Firmness

The firmness of the nanohydrogel-coated and uncoated fresh-cut apples was evaluated according to the method suggested by Abugoch et al. [38]. Herein, a texture analyzer (AMETEK Brookfield CT3, Tokyo, Japan) was used to determine the firmness of the samples. In this investigation, an apple slice of 22 mm was penetrated to a depth of 15 mm at a consistent rate of 15 mm/s using a 9 mm diameter probe that applied a regulated penetrating force. Four fresh-cut apples, two coated and two uncoated, were chosen at random for the experiment on the 0th, 2nd, 4th, 6th, 8th, and 10th days. 

#### 4.4.4. Antioxidant Activity

The antioxidant activity was assessed by determining the free radical scavenging effect (2,2-diphenyl-1-picryl-hydrazyl (DPPH)) of uncoated fresh-cut apples and those coated with nanohydrogel using the method suggested by Ganiari et al. [39]. Briefly, cut apples (25 g each coated and control) were added to 100 mL of distilled water, and slurry was prepared using a laboratory hand blender. The slurry of control and coated cut apples was centrifuged at 6000 rpm for 20 min, and 200 µL of the supernatant was added into 7.8 mL methanolic DPPH solution (0.050 g/L). The sample was vortexed and placed in the dark for 30 min. The absorbance of the sample was measured using a UV-visible spectrophotometer (Manti Lab solutions, Panchkula, India) at 517 nm wavelength, which was then reported as the percentage (%) of DPPH radical inhibition and calculated using the following equation.

(2) $$Antioxidant\;activity\left(\%\right)=\left[\left(\left(\frac{Absorbance\;of\;sample-Absorbance\;of\;control}{Absorbance\;of\;sample}\right)\times 100\right)\right]$$

#### 4.4.5. Vitamin C

The evaluation of the ascorbic acid (vitamin C) content was conducted using spectroscopic method described by Robles-Sánchez et al. [40]. Control and coated apples (20 g each) were homogenized in 100 mL of oxalic acid (H_2_C_2_O_4_) solution using a high-speed homogenizer at 5000 rpm for 5 min. The homogenized sample (80 mL) was filtered through Watman filter paper 1 and further diluted with 120 mL of 8% oxalic acid. Cupric sulfate (CuSO_4_) solution was added to an aliquot and again diluted with prepared oxalic acid. The absorbance of the sample was taken at 249 nm using a spectrophotometer (Raj, enterprise, Mumbai, India). The amount of vitamin C in 100 g of fresh-cut apples was calculated and expressed in milligrams (mg). 

#### 4.4.6. Respiration Rate

The respiration rate of the control and coated samples was determined based on headspace gas composition (CO_2_) method followed by Tosif et al. [13]. Briefly, control and coated samples (25 g each) were kept in a 500 mL airtight glass jar at room temperature (27 °C) for 24 h. During the 10 days of storage, samples of headspace were regularly checked and analyzed for CO_2_ content using gas chromatography (Hitachi Model 163). The gas chromatograph was equipped with a thermal conductivity detector and a CTR 1 column from Alltech Associates, Inc. Helium functioned as the carrier gas, flowing at a rate of 50 mL min^−1^. The column temperature, detector temperature, and inlet temperature were set at 55 °C, 100 °C, and 55 °C, respectively. 

#### 4.4.7. Total Phenolic Content 

The total phenolic content (TPC) of the control and coated samples was evaluated by homogenizing 10 g of each sample with a mixture of 10 mL of 1 % HCl and 10 mL of 1 % methanol solution and then centrifuged at 10,000 rpm for 20 min. The supernatant was collected and used for the determination of phenolic content. The concentration of the phenols in the control and coated apples was determined according to Folin–Ciocalteu assay using gallic acid as a standard, and all the results of the TPC were expressed as milligrams of gallic acid equivalent (GAE) per one kilogram of sample. Briefly, 250 µL of the sample diluted 51 times with double-distilled water was mixed with 2 mL of 10-fold diluted Folin–Ciocalteu reagent. After 10 min of equilibrium, 4 mL of 25% anhydrous sodium carbonate solution was added to the mixture and shaken thoroughly. After incubation for 20 min at ambient temperature, the blue color was developed, and absorbance was measured at 670 nm using a UV-visible spectrophotometer (Raj, enterprise, Mumbai, India).

#### 4.4.8. Microbial Analysis

Microbiological analysis was carried out in accordance with the protocol described by Valdes et al. [41] with the objective of quantifying yeast and mold counts, total psychrotrophic counts, and aerobic plate counts. For both coated and nanohydrogel-uncoated fresh-cut apples, measurements were taken in triplicate at different time intervals (0, 2, 4, 6, 8, and 10 days). Coated and uncoated fresh-cut apples (50 g each) per treatment were homogenized in sterile stomacher bags to demonstrate the procedure. Then, coated and uncoated cut apples (30 g each) were homogenized and transferred to another stomacher bag and mixed with 100 mL of buffered peptone water. After homogenizing the mixture for 10 min, this diluent was used to prepare 10-fold dilutions. For the microbiological counts, particular Petri dishes were used. 3M yeast and mold count plates and 3M aerobic plate count (APC) plates were used for the yeast and mold counts and aerobic counts, respectively. To find the aerobic counts, the 3M APC plates were incubated for 48 h at 37 °C. The APC plates were incubated at 4 °C for 5 days in order to facilitate psychrotrophic counts, while the 3M yeast and mold count plates were incubated at 20 °C for 6 days. Colony-forming units (log CFU) per gram of the sample were calculated after the colonies were counted using digital colony counter (MH enterprises, India) followed by incubation. This experiment provides significant context for understanding the microbiological quality and storage safety of fresh-cut apples.

#### 4.4.9. Statistical Analysis

All the data from the experiment performed in this study were analyzed using SPSS software (Version 17). One-way variance analysis (ANOVA) was applied to check the significant and non-significant differences within the treatments. All the data were collected in triplicates for all experiments, and the obtained results were reported as the mean ± standard deviation. Microsoft Excel 2019 was used to calculate average values and standard deviation. 

## Figures and Tables

**Table 1:** Droplet size and zeta potential of different formulated taro mucilage nanohydrogels.

Taro Mucilage Concentration (%) *w*/*v*	Droplet Size (nm)	Zeta Potential (mV)
1	245.35 ± 0.58	−24.36 ± 0.38
2	211.48 ± 1.24	−15.79 ± 0.43
3	175.61 ± 0.92	−30.25 ± 0.94
4	199.76 ± 0.75	−12.81 ± 0.71
5	186.19 ± 0.37	−19.46 ± 0.37

Data shown are the means of three replicates ± standard deviation.

## Data Availability

The data are contained within the article.
